# Multi-scale analysis of the community structure of the Twitter discourse around the Italian general elections of September 2022

**DOI:** 10.1038/s41598-024-65564-6

**Published:** 2024-07-10

**Authors:** Lorenzo Federico, Ayoub Mounim, Guido Caldarelli, Gianni Riotta

**Affiliations:** 1 LUISS Data Lab, Viale Pola 12, 00198 Rome, Italy; 2 Department of Political Sciences, LUISS University, Viale Romania 32, 00197 Rome, Italy; 3 Department of Molecular Sciences and Nanosystems, Ca’ Foscari University of Venice, 30172 Venice, Italy; 4 European Centre for Living Technology, 30124 Venice, Italy; 5 Institute for Complex Systems, Consiglio Nazionale delle Ricerche, UoS Sapienza, 00185 Rome, Italy; 6 London Institute for Mathematical Sciences, W1K2XF London, UK; 7 Fondazione Futuro delle Città, Via Boccaccio 50, 50133 Firenze, Italy

**Keywords:** Information theory and computation, Statistical physics, thermodynamics and nonlinear dynamics

## Abstract

We perform a multi-scale analysis of the geometric structure of the network of X (Twitter at the time of data collection) interactions surrounding the Italian snap general elections of September 25th 2022. We identify within it the communities related to the major Italian political parties and after it we analyse both the large-scale structure of interactions between different parties, showing that it resembles the coalitions formed in the run-up to the elections and the internal structure of each community. We observe that some parties have a very centralised communication with the major leaders clearly occupying the central role, while others have a more horizontal communication strategy, with many accounts playing an important role. We observe that this can be characterized by checking whether the network in the community has a strongly connected giant component or not.

## Introduction

The use of Twitter (http://twitter.com) as a proxy of political tendencies and in particular as a quantitative way to assess the outcome of elections has recently become a typical case of study. The different expertise ranging from Political Sciences to Computer Science and Statistical Physics are needed in order to collect, analyse and make sense of the data. The Twitter microblogging platform has several limitations in downloading the data, and those limitations are becoming increasingly stricter with the passage to the new platform X. In this paper we shall still refer to Twitter (the old name) since this was the company when data were collected. Over the years many pieces of research focused on the collection and analysis of Twitter Data for the forecasting of outcomes. Examples range from the study of online movements^1^ to the study of elections ranging from Dutch elections^2^,to Italian^3^, Singapore^4^, France^5^ and US elections^6,7^, to name a few. The reasons for such a success lie in the fact that irrespectively of the obvious bias in the Twitter representation across different countries, and irrespectively of disorders of information, the signal present in Twitter is still very informative^8^. Twitter has the important characteristic of being both a medium for the official communication and campaigning by parties and individual politicians^9^ and for broader discussion among the public. For this importance in the shaping of the social consensus and for the impact that elections might have on society, there is a strong activity on Twitter and social media resulting in disorder of information^10^ and (on Twitter specifically) often relying on fake accounts often operated by automated software indicated by bots^11^. In more recent times this activity arrived even to support war propaganda^12^. In order to spot “bot-like” features various methods have been used. A first class is based both on the *semantic* of the messages retweeted, while a second one is based on the *topology*, *i.e.* the structure of the connections between the users. Some researchers have created an online analysis tool that can help quantify the probability of interacting with one bot (see for example https://botometer.osome.iu.edu). The problem has become so urgent that various political institutions have decided to launch various actions for transparency and increased commitment by the platforms in the verification of the news diffused^13^.

Having stressed the incredible efforts that people invested in turning on their side the sentiment of the users of social media, it should be now less surprising that the information present in the tweets can be used to forecast possible outcomes both political and financial^14,15^.

In this paper, we analyze the discussion on Twitter surrounding the Italian snap election of September 25th 2022 (from September 5th to October 2nd 2022). To do so we collected a large dataset of Tweets containing political keywords and used it to build a network of interactions between accounts (retweets and replies). The goal is to perform a multi-scale analysis of the network topology. The first step consists in detecting the largest communities in the network and identifying the political affiliations of their most important nodes. We find out that, as expected in similar studies (cfr e.g.^16^), communities largely correspond to political party affiliations, at least as far as official accounts of politicians and party institutions are considered. Every community also has among its more central nodes several media outlets and personalities, online influencers and even meme and satirical pages whose audiences are close to the party in question.

We then analyse both the internal geometry of each community, to understand the communication strategy of each party and the behavior of its followers and the inter-community structure, to see how it matches the left-right alignment of parties and the coalitions formed for the election. Finally we check the daily rates of appearance of edges over the period considered to see that, with the obvious exception of the election day itself, when Twitter engagement on political topics had an obvious upward spike, resemble log-normal distributions.

## Methods

### Data gathering

We extracted a dataset of around 5 million tweets using the Filter API from September 5th to October 2nd 2022. Tweets were collected searching for keywords and hashtags related to the Italian political landscape and filtered afterwards to be only in the Italian language, considering that some politically relevant terms (e.g. “CoViD”^17^ or “Putin”) were not Italy-specific. The full list of hashtags and keywords used in the search is too long to be included in the main body of the paper and provided as Supplementary material. Filter API (see^18^ for more detailed information) collects all the tweets satisfying the query as they are posted, thus avoiding the risk of missing tweets that are later removed, and has a rate limit corresponding to $1 \%$ of the total unfiltered volume of tweets posted. There was a loss of data for a blackout on September 23rd. We estimate the lost data to be around 2% of the total sample and do not expect it to have a relevant impact on the paper’s overall conclusion.

### The network structure to filter the interaction data

From the Twitter data, we built a network^19^ of interactions based on retweets and replies. The model was created as a directed network where the nodes are Twitter users and there is an oriented edge (*v*, *w*) if and only if user *v* has replied and retweeted at least once some (not necessarily the same) tweet from user *w*. The weight of the edge is the minimum between the number of retweets and replies from *v* to *w*. The network was built in this way to choose edges that represent both engagement (replies) and agreement (retweets) so that we expected the community structure to resemble the most relevant political formations and positions. The network has a total of 60469 directed edges (of which 1053 (self-)loops), with a total weight of 87569, over 24610 vertices.

## Results

### Connected components

We first analyzed the connected component structure, both weak and strong, of the network. A weakly connected component is a subgraph of the original network where all vertices are connected to each other by some path, ignoring the direction of edges. A strongly connected component is a subgraph where all ordered pairs of vertices are connected by some directed path^20^. There is a clear weakly connected giant component which covers the vast majority of the network. It is composed by 23069 vertices ($93.7\%$ of the total) and 59501 edges ($98.4\%$ of the total), all other components being very small, the second with only 10 vertices. The largest strongly connected component contains 1871 vertices, with the second largest being much smaller, with only 7 vertices in total. It is not surprising that the largest strongly connected component, while still qualifying as a giant component (*i.e.* a component that makes up a positive proportion of the network and is much larger than the second one), is much smaller compared to the weakly connected giant, given that, by definition, a vertex in the strongly connected giant component needs to have non-zero in-degree and out-degree and there are only 4449 such nodes. As we will show in “Degree sequence” section, the existence of a strongly connected giant component (and thus a weakly connected one) is to be expected given the degree distribution in the network.

### Degree sequence

**Figure 1:**
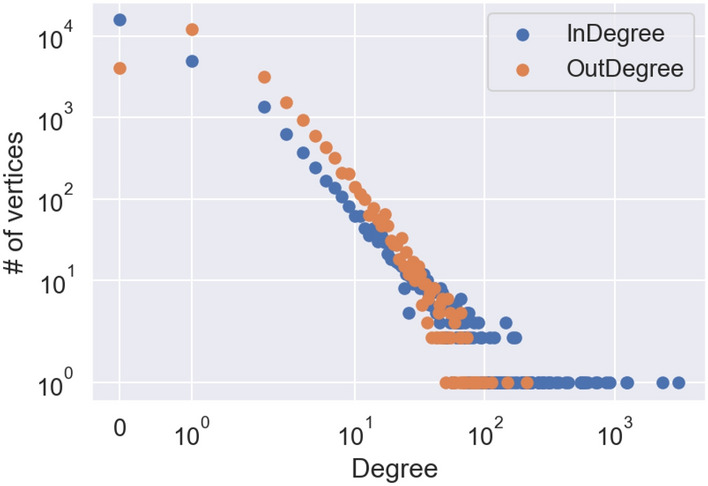
Log–log plot of the distributions of in- and out-degrees over the entire network.

We studied the distribution of the in- and out-degrees of a uniformly chosen node in the overall network. We recall that the in-degree of a user is the number of users that have bot retweeted and replied to them, while the out-degree is the number of users that they have both retweeted and replied to. As it is usually observed in social networks (see *e.g.*^21^, and as it has recently confirmed both experimentally^22,23^ and analytically^24^), both the sequences of in- and out-degrees follow a power-law distribution. This means that the number of vertices with in- or out-degree *k* decreases proportionally to $k^{-\gamma }$ for some $\gamma >0$. As we see, from Fig. 1, the in-degrees have heavier tail ($\gamma _{in}= 1.8473$) compared to out-degrees ($\gamma _{out}= 2.0467$). This is not surprising, considering that the political discussion is usually driven by a limited amount of very influential figures (as we see, usually the politicians themselves). They end up being vertices with very high in-degree, as their content receives a lot of engagement, but not necessarily high out-degree. The parameters of the power-laws are estimated using the Python library powerlaw.

The distribution of the in-degrees is extremely heavy-tailed, even for the standards of the scale-free distributions often observed in real-world networks (see e.g.^25^), the estimated exponent would imply the underlying distribution to have infinite mean. This might be explained by the fact that the network is sampled starting from the edges, and thus the selection of the vertices from the larger Twitter network happens in a size-biased way, that is, very active accounts are more likely to be part of the network we sampled than those with low or no activity.

**Figure 2:**
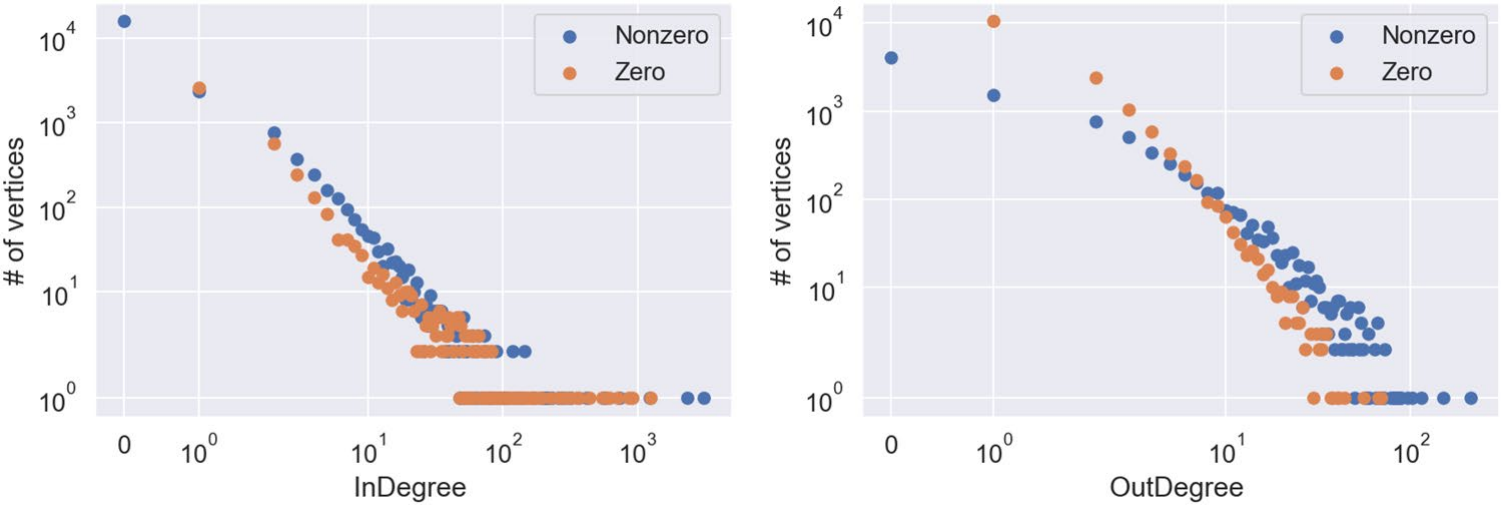
Log–log plot of in-degree separated between vertices with zero and nonzero out-degree (above) and of out-degree separated between vertices with zero and nonzero in-degree.

We also analysed the correlation between in- and out-degrees in the nodes of the network. We observe that in- and out-degrees are negatively correlated (Kendall $\tau =-0.148$, *p*-value = $1.16 10^{-162}$), but this is again an effect of the sampling of the network starting from the edges. In particular, in a network defined this way, a vertex with 0 in-degree necessarily has non-0 out-degree and vice versa, otherwise it would not have been picked up during the sampling. This artificially generates a negative correlation. If we restrict the analysis only to the vertices with both in- and out-degree at least 1 the correlation is instead positive (Kendall $\tau =0.201$, *p*-value = $8.56 10^{-70}$), as more active accounts, or accounts that are more focused on political themes, end up with higher both in and out-degree. As we see in Fig. 2, the distribution of out-degrees of vertices with non-zero in-degree is heavier-tailed than the one of the vertices with zero in-degree, but also, for the reason we explained, the second has no density at 0.

We can show that the the existence of a strongly connected giant component is a direct consequence of the degree distribution in the network. To do so, we analyze what the structure of a uniformly chosen graph with the same in- and out-degree distributions would look like. This is often done by building the so-called *hard configuration model.* In the directed version of the hard configuration model each vertex is given as many in-stubs as its in-degree and as many out-stubs as its out-degree. Then each in-stub in the graph is paired uniformly at random with an out-stub to form a directed edge in the network. For this procedure to be viable, it is necessary that the number of in-stubs is identical to the number of out-stubs, but this is trivially satisfied if we use as input the degrees of an observed network. The question of whether random digraphs have a giant strongly connected component, that, is whether for a sequence of random digraphs the proportion of nodes in the largest strongly connected components does not converge to 0 as the size of the graph increases, has been studied in depth, starting with the work of Karp in^26^. It was proved by Cooper and Frieze in^27^ that the existence of a giant strongly connected component in the typical network with a given degree sequence has a sharp phase transition, governed by the parameter

1 $$\begin{aligned} \nu = \frac{\mathbb {E}[D_{in}D_{out}]}{\mathbb {E}[D_{in}]}, \end{aligned}$$

where the duple $(D_{in},D_{out})$ is the in- and out-degree of a uniformly chosen random vertex. In particular, a giant strongly connected component is present with very high probability in networks with a specified degree sequence when $\nu >1$, while if $\nu <1$ the largest strongly connected component in the network has size at most proportional to the logarithm of the total number of vertices. In our network, we observe $\nu =4.861$, well above the phase transition critical threshold.

## Community structure

After analysing the macroscopic features of the graph, we zoom in to look for the mesoscopic community structure of the graph, trying to identify the different political groups and study the internal properties of each community. Our network was built in such a way that we expect most directed edges (*v*, *w*) to represent an expression of support from the account represented by *v* to the one represented by *w*. Consequently, we expect communities in the graph to correspond to political parties or anyway groups with coherent political positions.

### Community detection

To identify the communities relative to the various political parties, we tried several community detection algorithms in the weak giant component of the network. The one that gave us the best approximation of the real-world political groups was the “greedy modularity maximization”^28^, as implemented in greedy_modularity_communities from networkx. Greedy modularity maximization is a hierarchical clustering algorithm the partitions the network into disjoint communities. Initially each node is assigned to a community that contains only itself, and then sequentially communities are merged so to maximize at each step the *modularity function* of the partition *P*, described as

2 $$\begin{aligned} Q(P)=\frac{1}{2m}\sum _{i\in V(G)}\sum _{j \in V(G)}\Big (A_{ij}-r\frac{d_id_j}{2m}\Big )\delta _{P}(i,j), \end{aligned}$$

where *m* is the total number of edges in the network, $A_{i,j}$ is the number of edges between *i* and *j*, $d_i,d_j$ are the degrees of the nodes, that is, the number of nodes incident to them, $\delta _P(i,j)$ is the indicator of the event that *i* and *j* are in the same community, and *r* is the *resolution* parameter, which tunes how large we want the largest communities to be. For this network we tried different values of *r* and found that the best value to .

**Figure 3:**
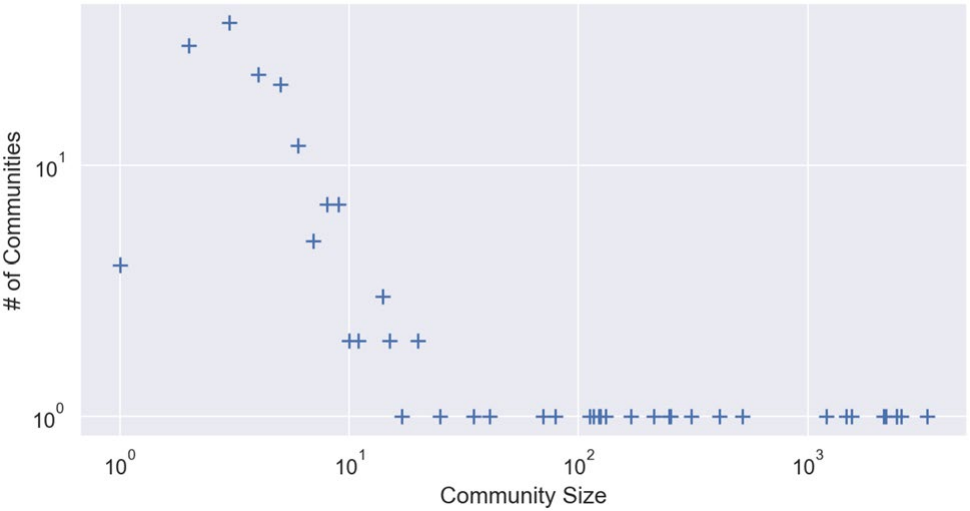
Log–log plot of the distribution of the size in terms of vertices of the communities found by the algorithm.

Greedy modularity is known (see e.g.^28^, Section 3) to produce communities with a distribution of the sizes that follows a power law, as shown in Fig. 3, except for a few particularly large communities (see Table 1. Plotting the distribution of the size of the 184 communities identified by the algorithm in Fig. 3, we see that this indeed happens in our data.

**Table 1:** Number of vertices and edges in the 10 largest communities.

	Vertices	Edges
Fratelli d’Italia	3309	3898
Italia Viva	2553	8000
Movimento 5 Stelle	2440	8389
Partito Democratico	2434	4216
Far Right	2201	4545
Lega	2150	5108
Azione	1549	1631
Anti-Establishment	1468	1920
Unione Popolare	1210	1874
Sinistra Italiana & Verdi	521	582

Here in Table 2 are the 10 most influential nodes (measured by their in-degree) in each of the 10 largest communities identified. Communities are named *a posteriori* based on their political alignment.

**Table 2:** The 10 vertices with the highest indegree in the 10 largest communities.

**Fratelli d’Italia**	**Italia Viva**	**Movimento 5 Stelle**	**Partito Democratico**	**Far Right**
GiorgiaMeloni	matteorenzi	GiuseppeConteIT	EnricoLetta	Account #1
FratellidItalia	marattin	Mov5Stelle	pdnetwork	Account # 2
GuidoCrosetto	Account #3	Account # 3	Account #3	Account # 3
berlusconi	ItaliaViva	Account #4	ellyesse	Account # 4
DSantanche	Account # 5	Account #5	pbersani	Account # 5
Account # 6	Account # 6	Account # 6	sbonaccini	Account # 6
isabellarauti	Account # 7	Account #7	serracchiani	Account # 7
zaiapresidente	Account # 8	Account #8	giorgio_gori	Account # 8
Account # 9	Account # 9	Account #9	Account #9	Account # 9
RaffaeleFitto	Account # 10	Account #10	lauraboldrini	Account # 10

**Lega**	**Azione**	**Anti-Establishment**	**Unione Popolare**	**Verdi e Sinistra**
borghi_claudio	CarloCalenda	ladyonorato	Account #1	Account #1
matteosalvinimi	Account #2	Account #2	Account #2	PossibileIt
AlexBazzaro	Account #3	gparagone	unione_popolare	Account #3
Account #4	msgelmini	Account #4	Account #4	Account #4
LegaSalvini	Account #5	Account #5	Account #5	Account #5
Account #6	Account #6	Account #6	Account #6	Account #6
AlbertoBagnai	Account #7	Account #7	Account #7	Account #7
Account #8	Account #8	Account #8	Account #8	Account #8
Account #9	Account #9	Account #9	Account #9	Account #9
Rinaldi_euro	Account # 10	Account #10	Account #10	Account #10

We see that there is a strong correspondence with major parties or political positions , even if not completely accurate, e.g. the Veneto Regional President Luca Zaia (Twitter handle *zaiapresidente*) is a member of the Lega and should appear in its community instead of the one of Fratelli d’Italia. This might be an inaccuracy, but might also be politically significant, as Zaia has been very critical of the Lega leadership during the period considered and thus it is expected that people who support him personally would not be supportive of Lega overall. The only party to make it above the $3\%$ threshold to win proportional representation seats at the elections which does not appear as its own community (mostly due to its sporadic presence on Twitter) is Forza Italia, with its leader Silvio Berlusconi (Twitter handle *berlusconi*) being put in the Fratelli d’Italia community, the biggest party of the center-right coalition they were part of. We further note the existence of two communities, which we called Far Right and Anti-Establishment, that lump together political figures from a multitude of smaller organizations characterized by radical right-wing and populist anti-EU sentiment respectively.

Here and there appear also accounts of media outlets and personalities and political satire/memes accounts which do not form a specific community, but are lumped together with the parties that are supported by the majority of their Twitter audience.

### Inter-community connections

**Figure 4:**
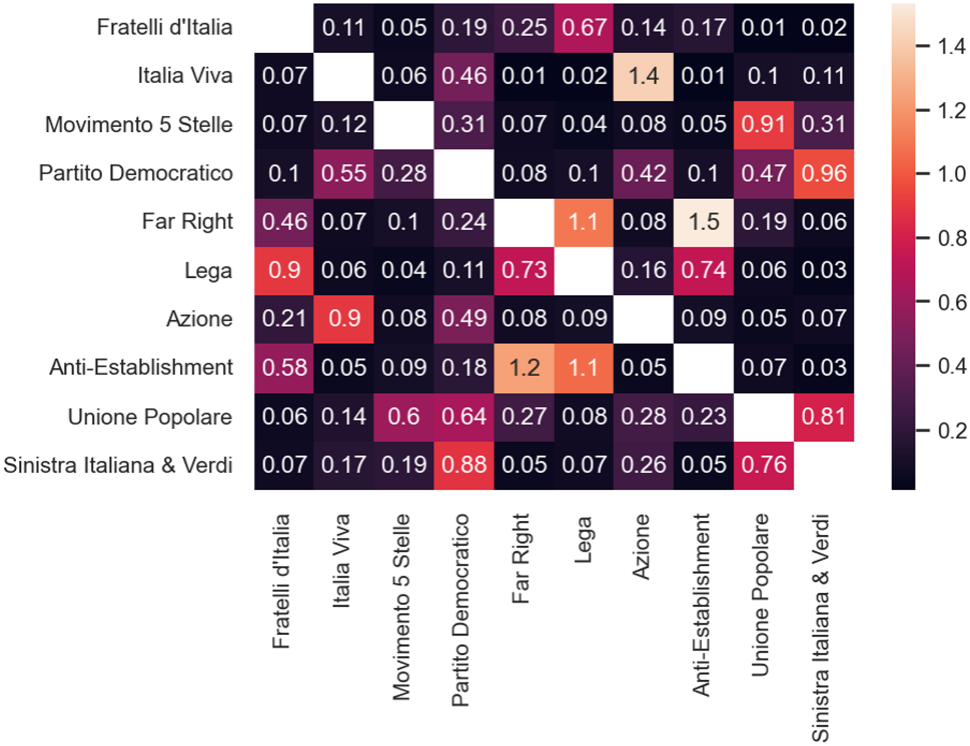
Heatmap of the values of $\gamma$ among the 10 largest communities.

**Figure 5:**
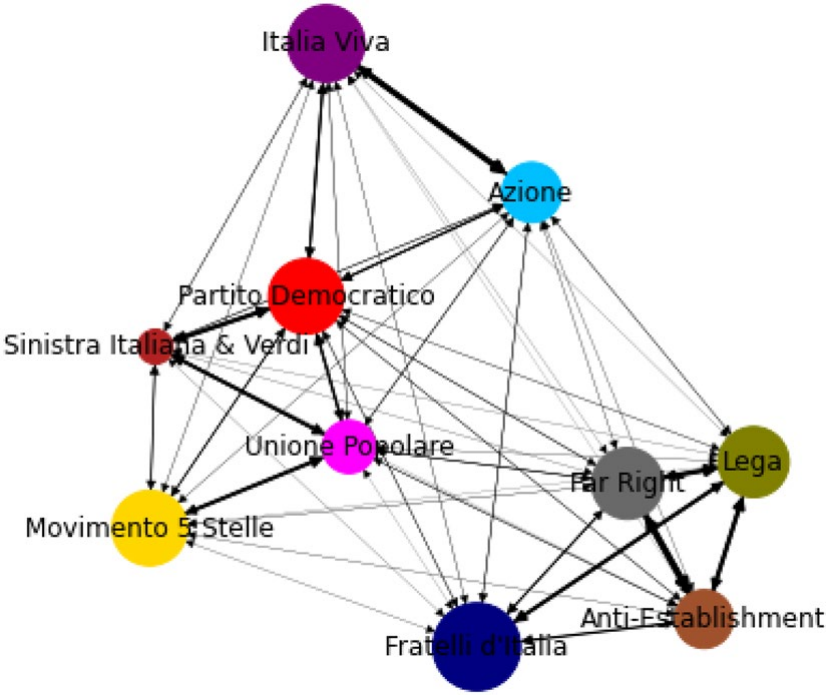
Network of inter-community connections. The width of the edges is given by $\gamma (C_i,C_j)$.

We also analyze the interconnections among the communities to see how the politicians of different parties and their followers interact with each other. We find that we can reconstruct quite accurately political coalitions from the inter-community connections. We compute for every couple of communities $C_i,C_j$ the variable

3 $$\begin{aligned} \gamma (C_i,C_j)=E(C_i,C_j)\frac{E}{E_{out}(C_i)E_{in}(C_j)}, \end{aligned}$$

where $E(C_i,C_j)$ is the number of edges starting from $C_i$ and ending in $C_j$, $E_{out}(C_i)$ is the total out-degree of the vertices in $C_i$, $E_{in}(C_j)$ is the total in-degree of the vertices in $C_j$ and *E* the total number of edges in the network. In particular, we are measuring the ratio between the number of edges actually present from community $C_i$ to community $C_j$ and its expected value in a null model like the configuration model. Since the communities were found via greedy modularity maximisation with resolution 2, $\gamma (C_i,C_j) \in [0,2]$, as the community detection algorithm would merge any communities for which $\gamma (C_i,C_j)>2$. This is confirmed by the values we obtained, as shown in Fig. 4. As expected, the highest values are found between the two components of the Terzo Polo (Italia Viva and Azione), which ran as one party in the elections. In the cluster of right-wing groups (Lega, Far Right, Anti-Establishment) in this second case what we observe is that Lega is the middle point between the more institutional right, represented by the Fratelli d’Italia cluster, which includes also the Forza Italia leader Silvio Berlusconi, and the more anti-system and extra-parliamentary far-right groups. Different, as shown in Fig. 5, is the structure of the Italian center-left parties. Here we see that the strongest connections form a line graph from PD to Sinistra Italiana (which is to be expected as the two parties ran in the same coalition), then Unione Popolare and finally Movimento 5 stelle.

### Intra-community interactions

We shall next analyse the structure of the induced subgraphs in the 10 largest communities. This would allow to quantify the differences in the behaviour of the various parties around the elections.

The internal structure of communities is also very interesting. In almost all cases we see just two different kinds of communities:

- **Top-down communities**, which have one or very few accounts with extremely high in-degree, and no account with high out-degree. These communities are mostly composed of one star or a few intersecting stars centered around their main leaders or influencers. We find 4 top-down communities among the 10 we studied: Fratelli d’Italia, Azione, Anti-Establishment and Sinistra Italiana & Verdi.
- **Broad discussion communities**, which instead have the internal distribution of both in- and out-degrees resembling a power law. Furthermore, the induced subgraphs have a giant strongly connected component inside. We find 6 broad discussion communities among the 10 we studied: Italia Viva, Movimento 5 Stelle, Partito Democratico, Far Right, Lega and Unione Popolare.

**Figure 6:**
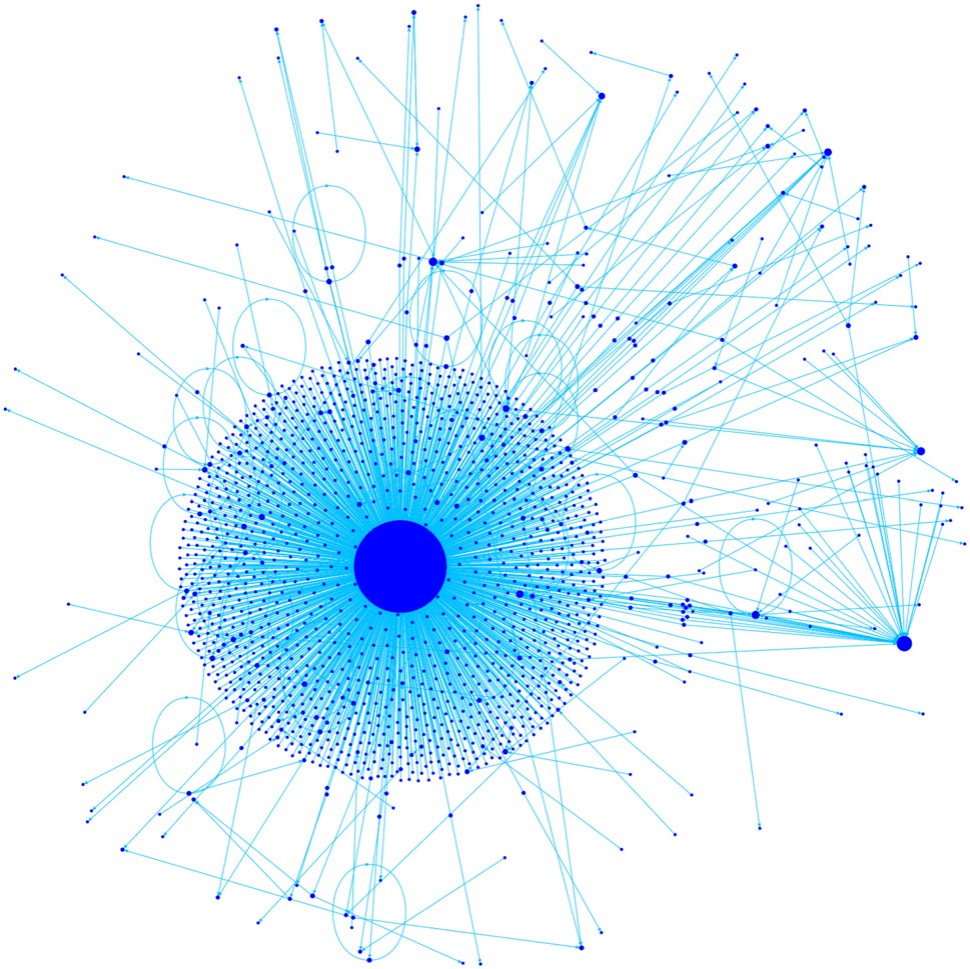
Network structure of the Azione community.

**Figure 7:**
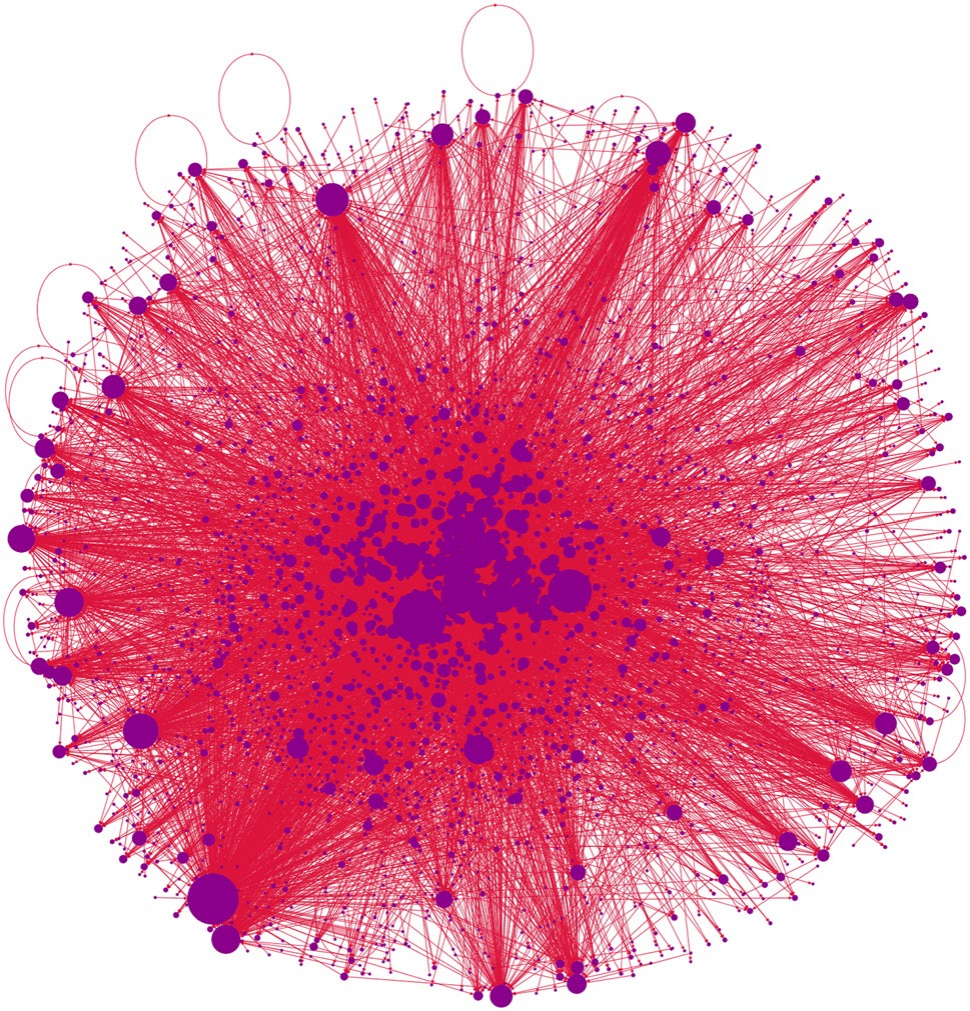
Network structure of the Italia Viva community.

While the difference in the structure of these communities is quite evident at first glance (see Figs. 6 and 7, we shall provide in the remainder of this section several ways to individuate the two groups. In particular, we will show in the upcoming sections, the existence of a giant strongly connected component in the subgraph induced by a community is a good way to differentiate them. We show here the difference between the two kinds of communities showcasing the two parties (Azione and Italia Viva) that formed the commonly called Terzo Polo centrist coalition.

#### Example of a top-down community: Azione

The Azione community is extremely centered around the figure of its political leader Carlo Calenda. Out of 1631 edges within the community, 1362 are pointed at Calenda. The maximum out-degree within the community is 5, and there is no giant strongly connected component (the largest has only 2 vertices). This a clear sign of the fact that he ran the campaign of the Terzo Polo very proactively, with a particular care for social media outreach.

#### Example of a discoursive community: Italia Viva

The other half of the Terzo Polo, Italia Viva, shows a very different picture. During the election campaign its leader, Matteo Renzi, took a step back in terms of both mainstream and social media presence. As a result, we see that the network community associated with Italia Viva has a very different structure, with multiple accounts with high in-degree, with Matteo Renzi himself barely leading (467) over Luigi Marattin (427). Also, we have accounts with quite high out-degree, up to 116, and a sizable strongly connected component, made of 247 nodes.

### Internal largest strongly connected component for the communities

We then analyse the structure of the strongly connected components in the induced subgraph for each of the 10 major communities. We remark that communities identified by greedy modularity maximization are by definition always weakly connected. We have noted, as reported in Table 3 that some of the communities have an internal giant strong component, with even hundreds of vertices, while some do not at all, with Fratelli d’Italia and Azione having no strongly connected component larger than 2 vertices. This gives us a clear distinction between Top-down and Broad Discourse communities, with Fratelli d’Italia, Azione, Anti-Establishment and Sinistra Italiana & Verdi in the first camp and Italia Viva, Movimento 5 Stelle, Partito Democratico, Far Right, Lega and Unione Popolare in the other.

**Table 3:** Size of the 2 largest strongly connected components and value of $\nu$ for the 10 largest communities.

	Largest	Second	$\nu$
Fratelli d’Italia	2	2	0.675
Italia Viva	247	7	6.633
Movimento 5 Stelle	399	2	6.905
Partito Democratico	50	7	1.455
Far Right	243	4	3.912
Lega	170	2	4.566
Azione	2	2	0.968
Anti-Establishment	5	2	0.759
Unione Popolare	69	7	2.574
Sinistra Italiana & Verdi	10	1	0.727

Such division is largely a direct consequence of the degree sequence inside the communities. To show this we explicitly compute for the subgraph induced by each community the parameter $\nu$ as defined in equation 1, which determines the existence of a giant strong component in a random graph with the given degree sequence, to formally separate the communities in two groups. For all the communities the parameter $\nu$ correctly predicts whether there is or not a giant strongly connected component.

It is important to be careful of the bias in this analysis due to the fact that networks built as communities in a larger network are necessarily weakly connected, while a random digraph with a given degree sequence is with high probability disconnected in the sparse regime.

### Internal degree structure of the communities

**Figure 8:**
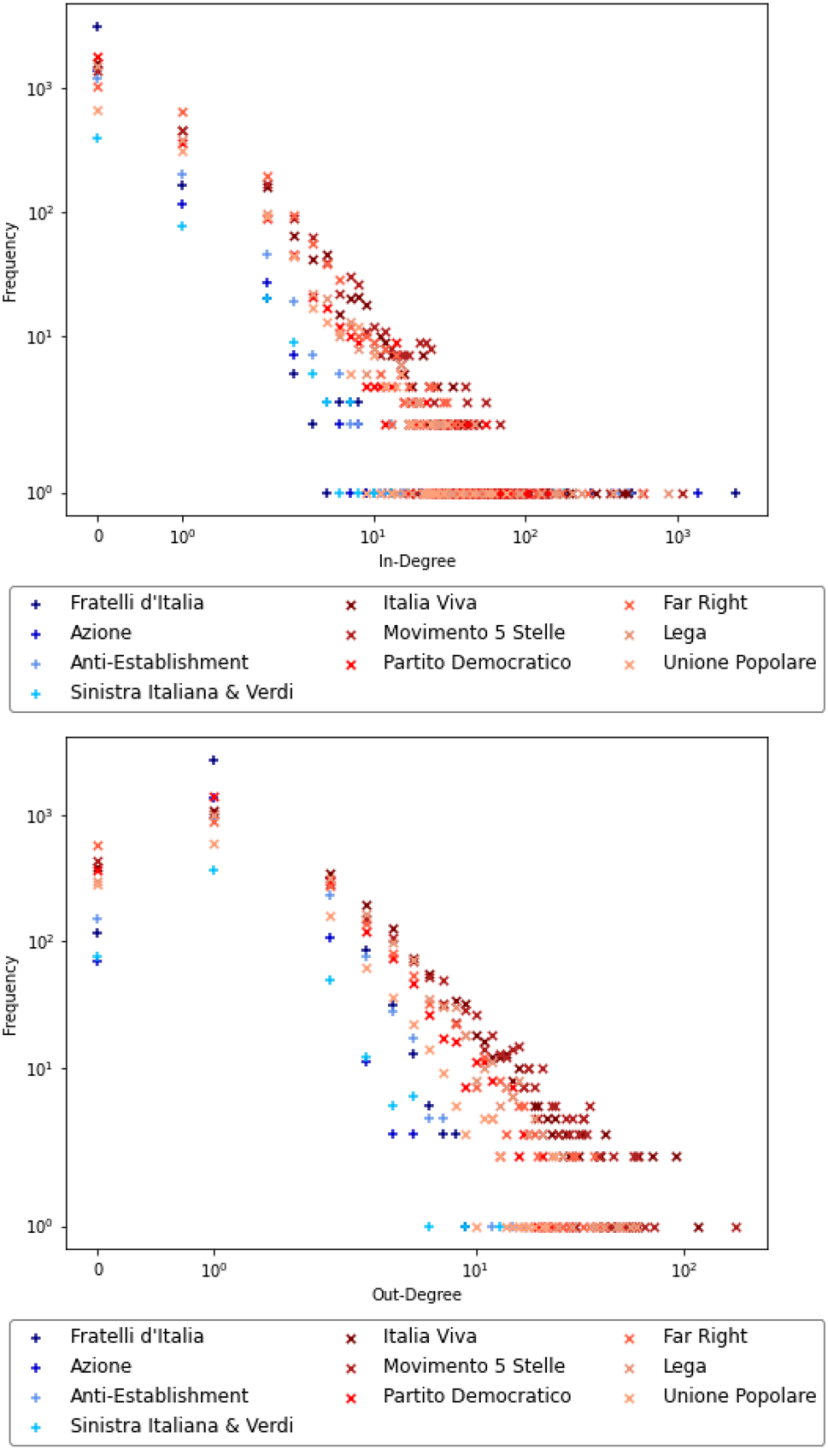
Log–log plots of the distributions of in-degrees (above) and out-degrees (below), shades of blue represent the top-down communities, shades of red the broad discussion communities.

We also analyse the degree sequences inside the subgraphs induced by the 10 communities. Also here we find that there is a clear separation between the two groups, as seen in Fig. 8. The Top-down communities have a much lighter-tailed out-degree distribution, with the maximum out-degree ranging from 5 (Azione) to 15 (Anti-Establishment) (see Table 4). The in-degree sequences are generally more light-tailed too, but with the presence of very few extreme outliers, which represent the main leaders and influencers and have in-degree orders of magnitude higher than all the other nodes. The broad discussion communities instead have a much heavier tail in the distribution of the out-degrees and a much more regular in-degree distribution, without such a clear separation between a few main leaders and an audience of followers.

**Table 4:** Maximum in- and out-degree among vertices in each community.

	Max in-degree	Max out-degree
Fratelli d’Italia	2400	9
Italia Viva	467	116
Movimento 5 Stelle	1075	177
Partito Democratico	588	28
Far Right	257	58
Lega	867	59
Azione	1362	5
Anti-Establishment	478	15
Unione Popolare	74	24
Sinistra Italiana & Verdi	178	13

## Time evolution of information flow among communities

**Figure 9:**
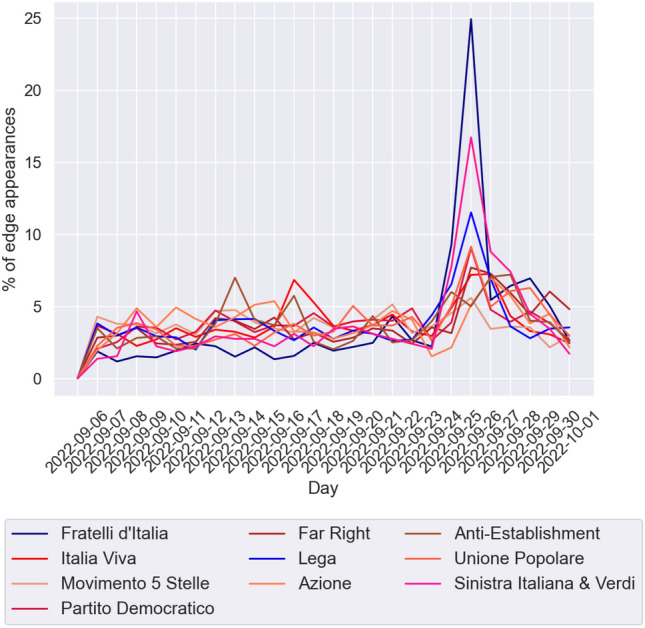
Plot of the time evolution of new edges within the same community. The *x* axis represents the day and the *y* axis is the proportion of the total edges that appeared on that day.

We finally study the time evolution of the network, in particular, we compute the distribution of the day in which edges appear in every community to see if the build-up of edges is the result of a time-independent process or if there are specific events (obviously the main candidate being the election itself) or individual tweets that produce on their own a significant proportion of the edges of a community. We recall that in the network we have defined the edge (*v*, *w*) in such a a way to indicate that account *v* has both retweeted and replied to account *w*. The time at which the edge appears is the the time when this condition is met, that is, the latest between the time of the first retweet from *v* to *w* and that of the first reply from *v* to *w*. Because the largest communities have a few thousand edges, we analyze the time evolution of the network on a daily scale, as shown in Fig. 9. Using a finer resolution on these data would result in many time intervals containing no new edges at all for the smaller communities, in particular during nighttime.

We test the hypothesis that the number of new edges that appear among vertices of the community is a sequence of independent identically distributed (iid) log-normal variables, that is, that, calling $E_d(C_i,C_j)$ the number of edges from community *i* to community *j* that have appeared in the *d*-th day of the time interval,

4 $$\begin{aligned} \log (E_d(C_i,C_i)) \sim N(\mu _i,\sigma _i)\quad \forall \ d. \end{aligned}$$

**Figure 10:**
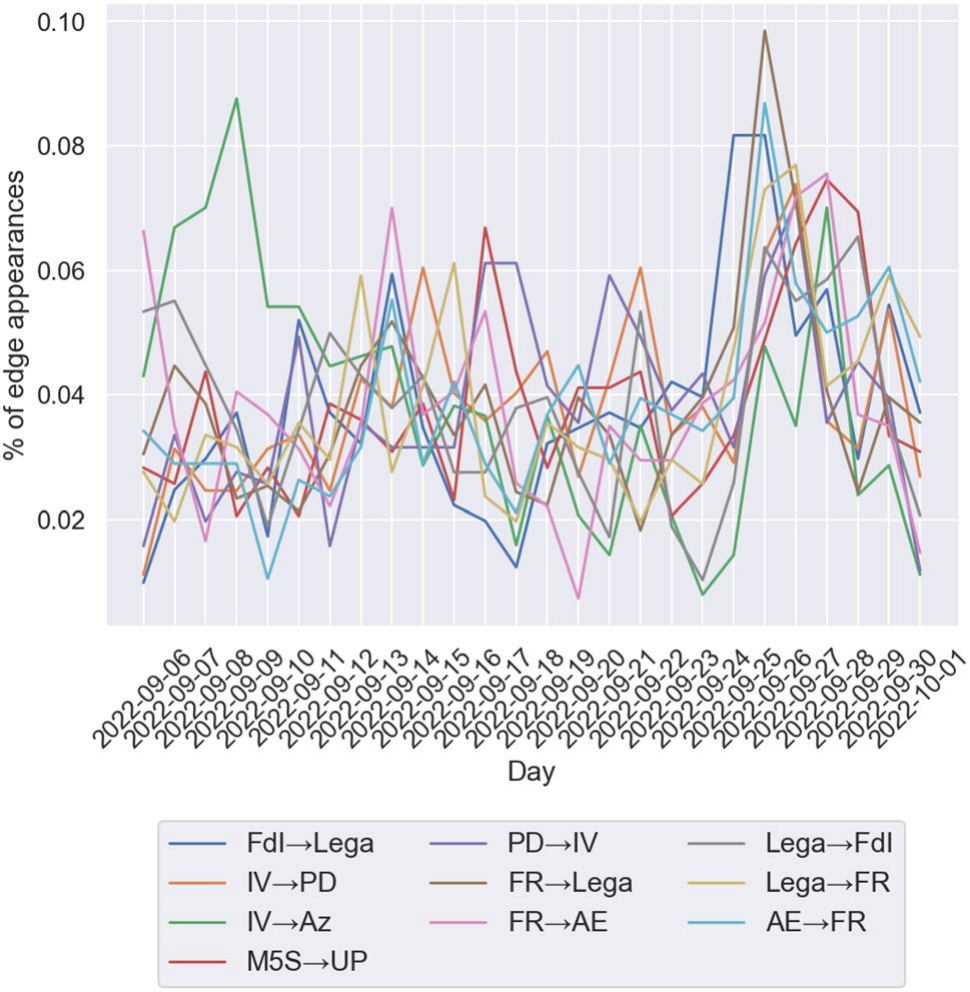
Plot of the time evolution of new edges across different communities. The *x* axis represents the day and the *y* axis is the proportion of the total edges that appeared on that day.

To do so, we apply the D’Agostino-Pearson Test^29^ to the sequence $(\log (E_d(C_i,C_i)))_{d \ge 1}$. What we observe is that, using a level of significance of $\alpha =0.05$, with Holm-Bonferroni correction^30^ for the 10 independent tests, so that the *k*-th smallest *p*-value $P_k$ is considered significant if $P_k< \alpha /(11-k)$. We observe that 2 communities out of 10 (Fratelli d’Italia and Lega) can reject the hypothesis that new edges appear daily as a sequence of iid log-normal variables, while Partito Democratico and Sinistra Italiana & Verdi would have rejected the null hypothesis in the uncorrected test (see Table 5). As expected, the main outlier in these cases is September 26th, the day after the elections, when the tweets from the most popular leaders of these parties got a huge amount of engagement. In Fratelli d’Italia’s case, as we have already shown, this engagement was mostly directed at their leader Giorgia Meloni, who, given the resounding success was widely expected to be nominated Prime Minister (she would eventually form the new government on October 22nd, 2022). It is worth noting that both distributions that are different from a log-normal are not so anymore if we remove the $E_d(C_i,C_i))$ for $d=21$, which corresponds to September 26th. We also ran the same analysis on the appearance of edges between different communities, as shown in Fig. 10. For many couples of communities $C_i,C_j$ the value of $E(C_i,C_j)$ is too small to perform any statistical analysis, so we focus on the 10 couples for which $E(C_i,C_j)$ is the highest. In this case, we observe that for no flow we are able to reject the hypothesis that $(E_d(C_i,C_i))_{d \ge 1}$ are iid log-normal variables (see Table 6).

**Table 5:** Mean, variance and results of the normality test for the logarithm of the number of new edges that appear daily in each community. Statistically significant *p*-values are in bold charachters.

	$\mu$	$\sigma ^2$	Statistic	*p*-value	H-B threshold
Fratelli d’Italia	4.675256	0.495916	13.235	**0**.**001337**	0.0056
Italia Viva	5.671632	0.097602	4.973	0.083180	0.0083
Movimento 5 Stelle	5.752756	0.039597	2.998	0.223370	0.0167
Partito Democratico	5.038250	0.084606	7.277	0.026285	0.0071
Far Right	5.097529	0.111467	3.120	0.210173	0.0125
Lega	5.199230	0.128604	17.369	**0**.**000169**	0.0050
Azione	4.071137	0.137161	1.616	0.445714	0.0500
Anti-Establishment	4.221723	0.157432	2.130	0.344732	0.0250
Unione Popolare	4.198808	0.122598	3.517	0.172297	0.0100
Sinistra Italiana & Verdi	2.904688	0.335514	9.351	0.009319	0.0063

**Table 6:** Mean, variance and results of the normality test for the logarithm of the number of new edges that appear daily between communities.

	$\mu$	$\sigma ^2$	Statistic	*p*-value	H-B threshold
FdI$\rightarrow$Lega	2.631294	0.254656	2.766	0.250845	0.0125
IV$\rightarrow$PD	2.776744	0.150457	5.972	0.050491	0.0056
IV$\rightarrow$Az	3.025617	0.374936	1.866	0.393313	0.0167
M5S$\rightarrow$UP	2.635332	0.141056	1.052	0.590935	0.0500
PD$\rightarrow$IV	2.882876	0.201654	2.967	0.226896	0.0100
FR$\rightarrow$Lega	3.561058	0.142449	3.041	0.218586	0.0083
FR$\rightarrow$AE	2.925962	0.269467	7.724	0.021028	0.0050
Lega$\rightarrow$FdI	3.015072	0.214907	3.620	0.163623	0.0071
Lega$\rightarrow$FR	2.892934	0.156091	1.446	0.485374	0.0250
AE$\rightarrow$FR	2.605695	0.167980	5.817	0.054568	0.0063

## Conclusions

In this paper, we built a network of Twitter interactions surrounding the Italian general elections of September 25th 2022. Such network is based on a large dataset of 5 million tweets in the Italian language related to politically relevant keywords for a month around election day. The vertices of the network are the Twitter accounts posting on elections and the directed edges represent the presence of both retweets and replies during the period considered. We analysed the community structure of the network and recovered a community structure that largely resembles the actual official political affiliations. We observe that there is a hierarchical structure. The communities identify mostly single political parties, with few exceptions: the very few relevant accounts related to Forza Italia were grouped together with Fratelli d’Italia, many small Far Right organizations were grouped together, and some Anti-Establishment populist groups (mainly Italexit and Italia Sovrana e Popolare) also forming a single community. But if we look at the macroscopic structure of the network that is, the interconnections between the communities representing different parties we find, as expected, a left-right split, but also a non-trivial internal geometric structure of the coalitions. We zoomed inside each of the communities to understand the social communication patterns followed by the different political parties, finding that they broadly divide into two groups. Some of them have a highly centralized communication strategy in which one or very few political leaders account for the majority of the impact, that is, the vast majority of the edges in the community point towards them. This is the most pronounced in the case of Azione, with its leader, Carlo Calenda, accounting for more than 80% of the total in-degree. Others on the other hand have a more horizontal way of communicating, with many different accounts having relatively high in-degree, and also with some accounts having large out-degree, sign that they engage with a lot of different political figures within the party on Twitter. The main discerning factor between the two groups is that in the first case there is no giant strongly connected component within the community found on the network, as the networks largely resemble one or a few stars around the major accounts, and the vast majority of nodes have 0 in-degree. Finally, we analysed the time evolution of the daily appearance of new edges in the network during the period considered. What we found is that the number of new edges within a community or between two specific communities resembles a log-normal law, with an expected spike in the number of new edges on the day of the election itself, in particular for the parties of the winning coalition.

### Supplementary Information

Supplementary Information.

Supplementary Figures.

## Data Availability

The corresponding author will provide the IDs of the tweets to be hydrated with the API of X and the custom code developed for the analysis upon reasonable request.
