# Author Correction: Multi-scale analysis of the community structure of the Twitter discourse around the Italian general elections of September 2022

**DOI:** 10.1038/s41598-024-68911-9

**Published:** 2024-08-05

**Authors:** Lorenzo Federico, Ayoub Mounim, Guido Caldarelli, Gianni Riotta

**Affiliations:** 1 LUISS Data Lab, Viale Pola 12, 00198 Rome, Italy; 2 Department of Political Sciences, LUISS University, Viale Romania 32, 00197 Rome, Italy; 3 Department of Molecular Sciences and Nanosystems, Ca’ Foscari University of Venice, 30172 Venice, Italy; 4 European Centre for Living Technology, 30124 Venice, Italy; 5 Institute for Complex Systems, Consiglio Nazionale delle Ricerche, UoS Sapienza, 00185 Rome, Italy; 6 London Institute for Mathematical Sciences, London, W1K2XF UK; 7 Fondazione Futuro delle Città, Via Boccaccio 50, 50133 Firenze, Italy

Correction to: *Scientific Reports* 10.1038/s41598-024-65564-6, published online 10 July 2024

In the original version of this Article, the full list of hashtags and keywords used in the query to the Filter API for streaming search from September 5th to October 2nd 2022, was omitted from the Supplementary Information section.

This Supplementary Information file now accompanies the original Article.

