# Supplementary material for: Multi-scale analysis of the community structure of the Twitter discourse around the Italian general elections of September 2022
Source: Sci Rep. 2024 Jul 10;14:15980. doi: 10.1038/s41598-024-65564-6 (PMC11237110; doi:10.1038/s41598-024-65564-6)
Supplement: Supplementary file 1 — Supplementary Information. [file 41598_2024_65564_MOESM1_ESM.docx]

**Full list of hashtags and keywords used in the query to the Filter API for streaming search from September 5th to October 2nd 2022**

#25settembrevotolega,#credo,#italiasulserio,#salvini,#elezionipolitiche2022,#votafdi,#terzopolo,#meloni,#draghi,#italexit,#25settembre,#dammiil5,#dallapartegiusta,#elezioni,#noimoderati,#skytg24,#flattax,#scegli,#lega,#controcorrente,#agorarai,#tg2post,#liberidiscegliere,#fratelliditalia,#lavoro,#zonabianca,#centrodestra,#alleanzaverdisinistra,#pronti,#unionepopolare,#elezionipolitiche22,#pieroangela,#letta,#pd,#udc,#berlusconi,#azione,#pnrr,#gas,#carobollette,#luigisbarra,#calenda,#salariominimo,#lariachetira,#italia,#raccoltafirme,#inonda,#tgcom24,#milano,#fdi,#bombardieri,#omnibusla7,#morningnews,#elezioni2022,#venezia,#energia,#25settembrevotopd,#rainews24,#radioanchio,#liguria,#radiocusanotv,#covid19,#coraggioitalia,#vinconoleidee,#scuola,#s48,#ue,#radiolibertà,#goofynomics,#tg4,#forzaitalia,#mattarella,#europa,#agi,#ucraina,#firenze,#putin,#meeting22,#lacorsaalvoto,#ritornoalfuturo,#bologna,#governo,#giovani,#campagnaelettorale,#gorbaciov,#ferragosto,#russia,#pontemorandi,#greenpass,#lampedusa,#la7,#genova,#coffeebreak,#alikaogorchukwu,#redditodicittadinanza,#impegnimantenuti,#marcinelle,#crisanti,#rtl1025,#reneweurope,#radio24,#civitanovamarche,#24mattino,#stragedibologna,#covid,#cdx,#caroenergia,#bollette,#m5s,#diritti,#sicurezza,#frajese,#sanità,#giovanichenonhannovogliadilavorare,#dimaio,#bonuspsicologo,#versiliana,#unomattina,#speranza,#presidenzialismo,#conte,#blocconavale,#2agosto,#14agosto,#volontaripd,#tgla7,#tg1,#renewitalia,#politica,#legagiovani,#fratoianni,#elezionianticipate,#sfruttamento,#rete4,#orban,#mezzorainpiu,#lamorgese,#ionondimentico,#giorgiameloni,#gasparritour,#direzionepd,#difendiamoilmadeinitaly,#ambiente,#toscanasulserio,#sicilia,#piueuropa,#nato,#madeinitaly,#iovotoitaliaviva,#bastasbarchi,#wartsila,#toscana,#tg5,#obbligovaccinale,#novax,#meetingrimini,#medvedev,#lombardia,#fiom,#devianze,#comesivota,#aborto,#20e30,#tg2,#start,#senato,#roma,#puglia,#giustizia,#ghedini,#fuorisede,#famolo,#cgil,#cernobbio2022,#quota41,#portaaporta,#piùassumimenopaghi,#mezzorainpiù,#kosovo,#familyact,#campagnaelettorale2022,#agendadraghi,#25settembrevotoforzaitalia,#tasse,#piacenza,#patrimoniale,#maltempo,#italia2027,#iovotopiueuropa,#donne,pdnetwork,deputatipd,senatoripd,nzingaretti,enricoletta,liaquartapelle,andreaorlandosp,peppeprovenzano,itinagli,annaascani,serracchiani,simonabonafe,zanalessandro,pierofassino,sbonaccini,gdnazionale,pbersani,nomfup,guerini_lorenzo,andreamarcucci,graziano_delrio,darionardella,micheleemiliano,agora_dem,bartolopietro1,giorgio_gori,pcpadoan,tnannicini,monicacirinna,antonio_decaro,emanuelefiano,paola_demicheli,brandobenifei,mariannamadia,lottiluca,valeriafedeli,vincenzodeluca,robertapinotti,matteolepore,paologentiloni,pierferdinando,mov5stelle,giuseppeconteit,ale_dibattista,virginiaraggi,beppe_grillo,eliolannutti,carlosibilia,nicolamorra63,paolatavernam5s,m5s_senato,c_appendino,m5s_europa,azzolinalucia,roberto_fico,dellorco85,val_ciarambino,antolari1986,vitocrimi,paolapisano77,m5sroma,m5s_camera,lombard5stelle,sicilia5stelle,piemonte5stelle,mariadomenicac4,alfonsobonafede,legasalvini,matteosalvinimi,borghi_claudio,alexbazzaro,albertobagnai,rinaldi_euro,fontana3lorenzo,lega_gruppoid,massimocasanov3,legaregionelomb,legalombardasp,andreaostellari,lauraravetto,lega_senato,legacamera,claudiodurigon,fontanapres,susannaceccardi,volpi_raffaele,zaiapresidente,m_fedriga,molinaririk,sardonesilvia,giuliocentemero,senborgonzoni,amorellimilano,maxromeomb,simopillon,italiaviva,matteorenzi,marattin,lucianonobili,meb,davidefaraone,ivanscalfarotto,michele_anzaldi,ettore_rosato,francescobonif1,elenabonetti,teresabellanova,bobogiac,sandrogozi,radioleopoldait,marcodimaio,luigidimaio,piersileri,iolandadstasio,primodinicola,danieladonno,sergio_vaccaro1,cosimo_adelizzi,aleamitrano,battellisergio,v_casa_camera,lacastelli_it,federicadaga,manliods,f_duva,lucafrusone,chiara_201181,fratelliditalia,giorgiameloni,ignazio_larussa,guidocrosetto,dsantanche,giov_nazionale ,luciomalan,alberto_cirio,marcomarsilio,fidanzacarlo,fabiorampelli,donzelli,raffaelefitto,francescolollo1,isabellarauti,adolfo_urso,forza_italia,antonio_tajani,berlusconi,fi_giovani,liciaronzulli,antoniopalmieri,giorgiomule,gasparripdl,calabriatw,molesgiuseppe,berniniam,pres_casellati,flaviotositw,azione_it,carlocalenda,matteorichetti,pastorellagiu,enrico__costa,filippo_rossi,msgelmini,mara_carfagna,giovannitoti,gparagone,luigibrugnaro,maurizio_lupi,ladyonorato,renatobrunetta,l_cesa,udcita,alemannotw,distefanotw,mariodinolfi,casapounditalia,coraggio_italia,cambiamocontoti,ancora_italia,riconquistareit,stefanofassina,lauraboldrini,pietrograsso,robersperanza,possibileit,ellyesse,marcorizzopc,civati,aboubakar_soum,bentivoglimarco,cgilnazionale,uilofficial,cislnazionale,fiomnet,brunotabacci,antonioingroia,potere_alpopolo,articolounomdp,sinistrait_,partitcomunista,direzioneprc,piu_europa,emmabonino,riccardomagi,bendellavedova,cottarellicpi
