# Supplementary material for: Multi-scale analysis of the community structure of the Twitter discourse around the Italian general elections of September 2022
Source: Sci Rep. 2024 Jul 10;14:15980. doi: 10.1038/s41598-024-65564-6 (PMC11237110; doi:10.1038/s41598-024-65564-6)
Supplement: Supplementary file 2 — Supplementary Figures. [file 41598_2024_65564_MOESM2_ESM.pdf]

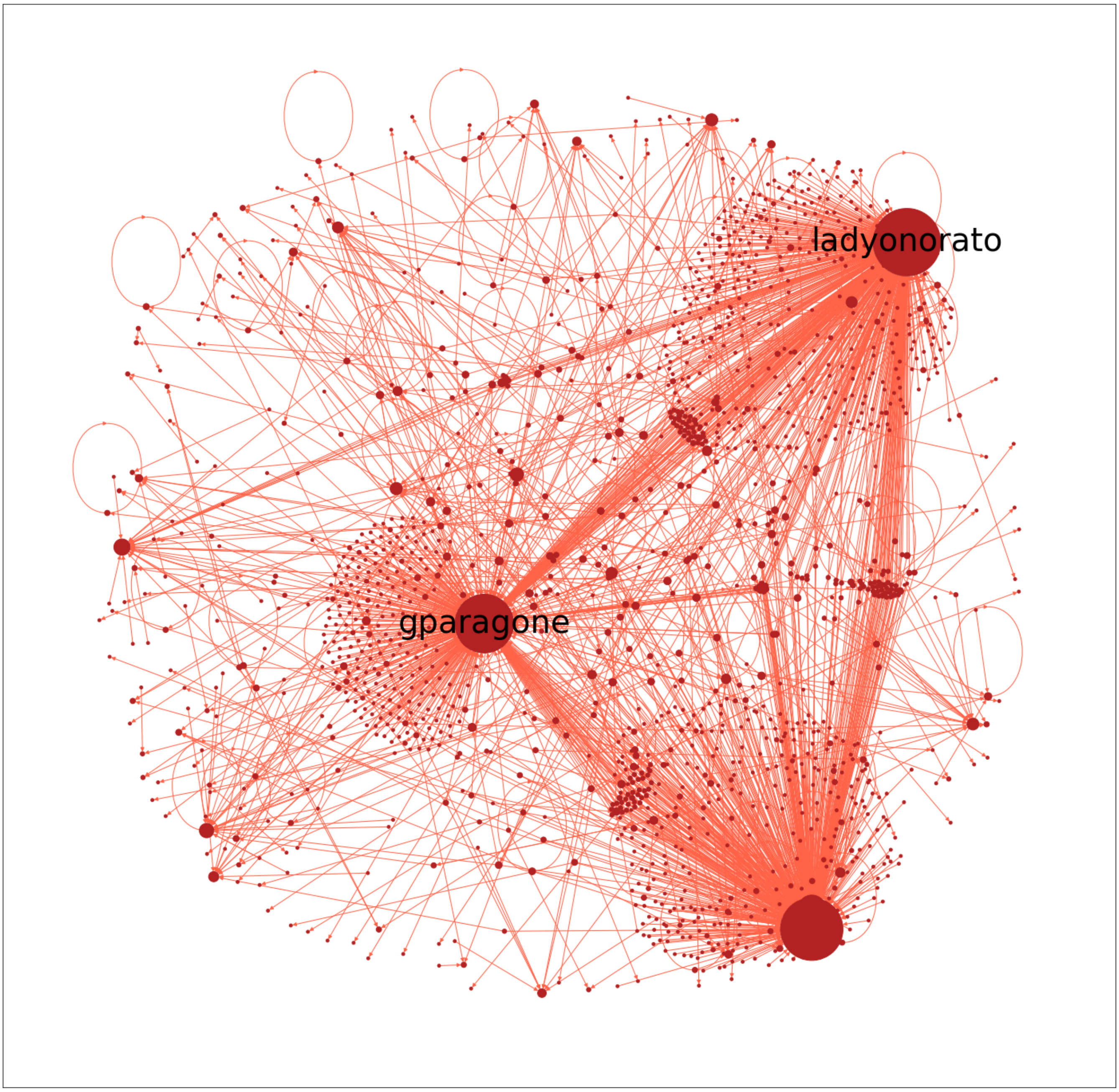

Figure 1: Plot of the Anti Establishment community. Vertex size is proportional to its in-degree, named accounts are those among the 10 with highest in-degree which are verified and belong to official party organs or affiliated politicians.

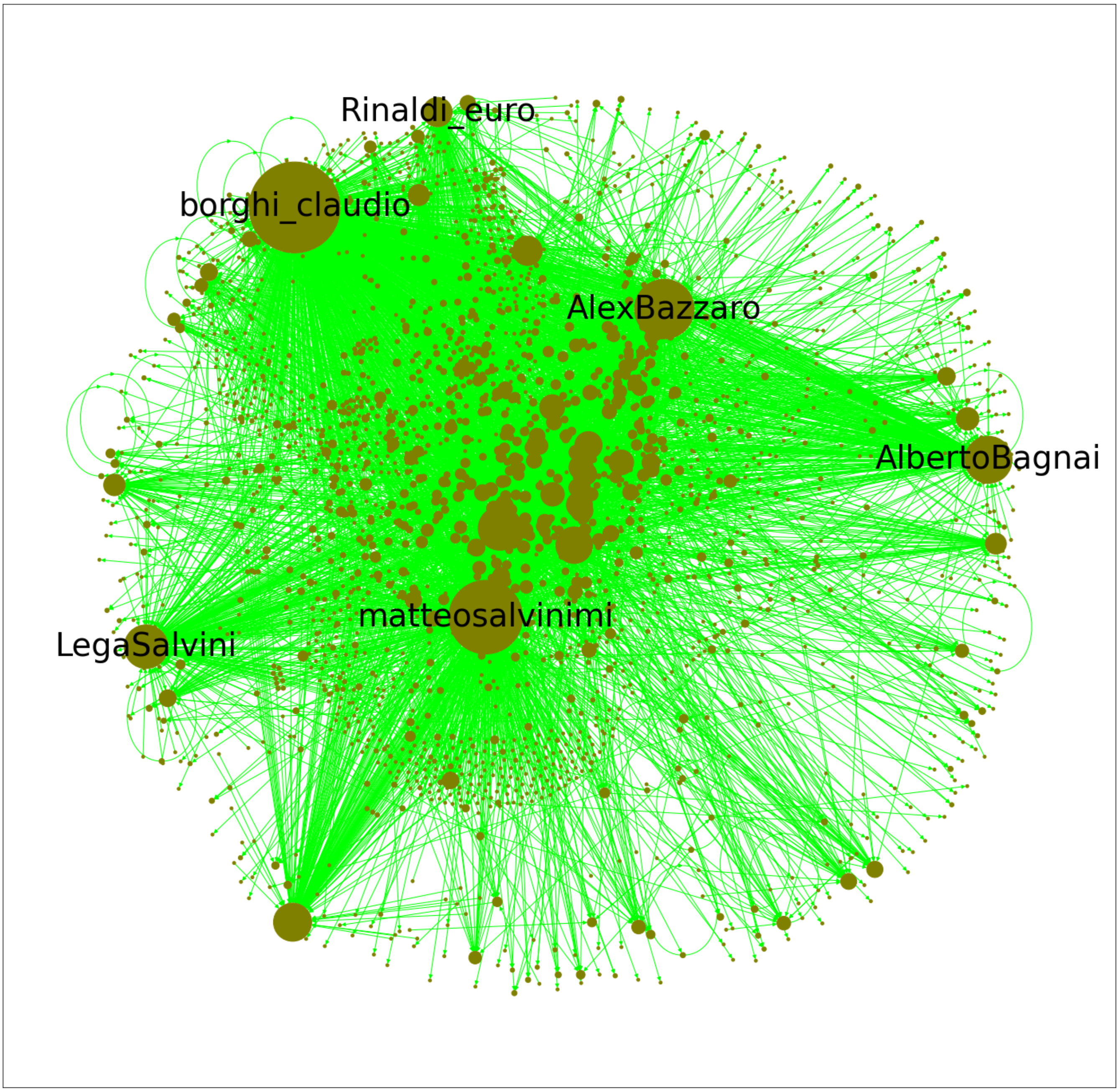

Figure 2: Plot of the Lega community. Vertex size is proportional to its in-degree, named accounts are those among the 10 with highest in-degree which are verified and belong to official party organs or affiliated politicians.

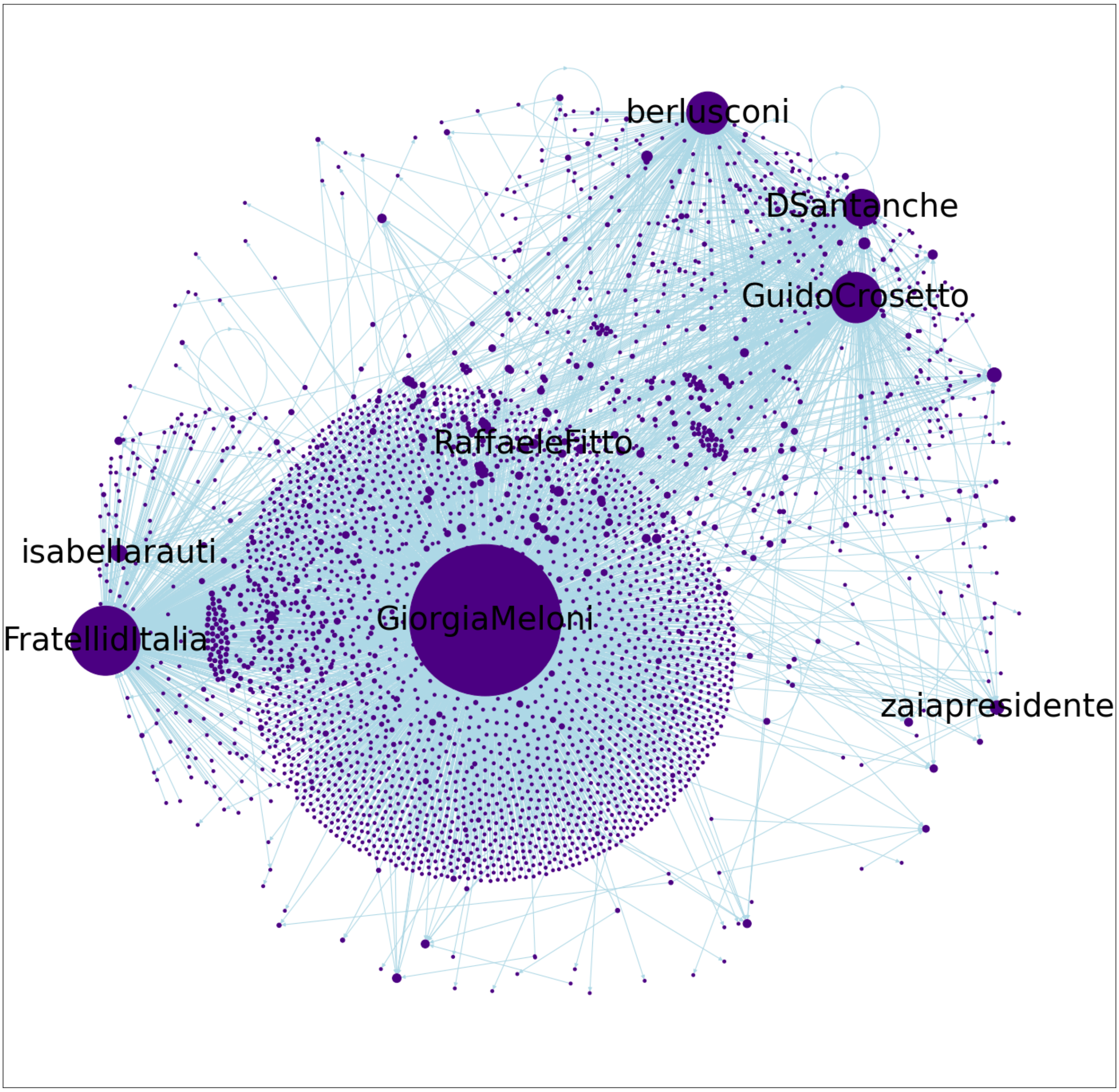

Figure 3: Plot of the Fratelli d'Italia community. Vertex size is proportional to its in-degree, named accounts are those among the 10 with highest in-degree which are verified and belong to official party organs or affiliated politicians.

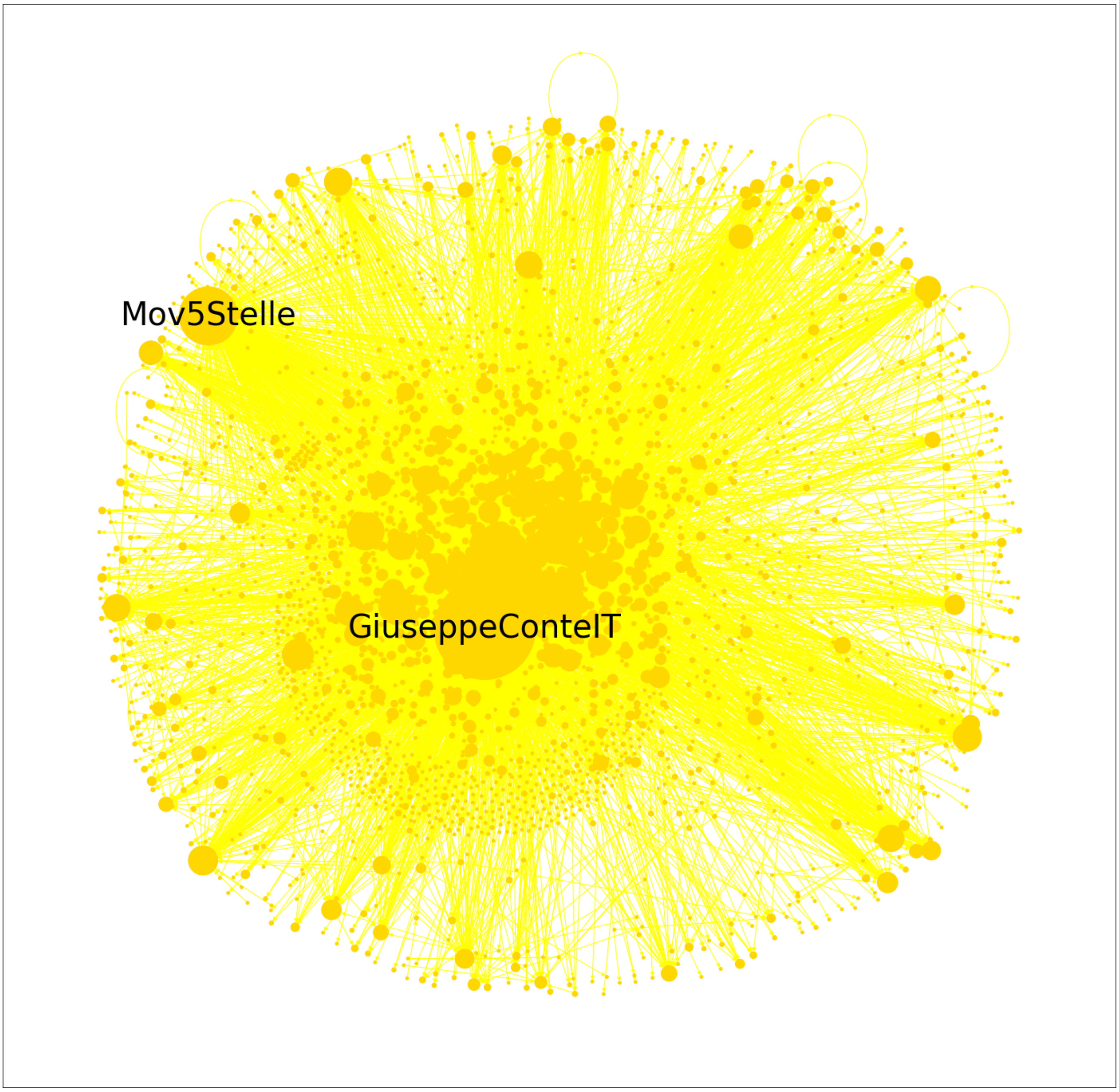

Figure 4: Plot of the Movimento 5 Stelle community. Vertex size is proportional to its in-degree, named accounts are those among the 10 with highest in-degree which are verified and belong to official party organs or affiliated politicians.

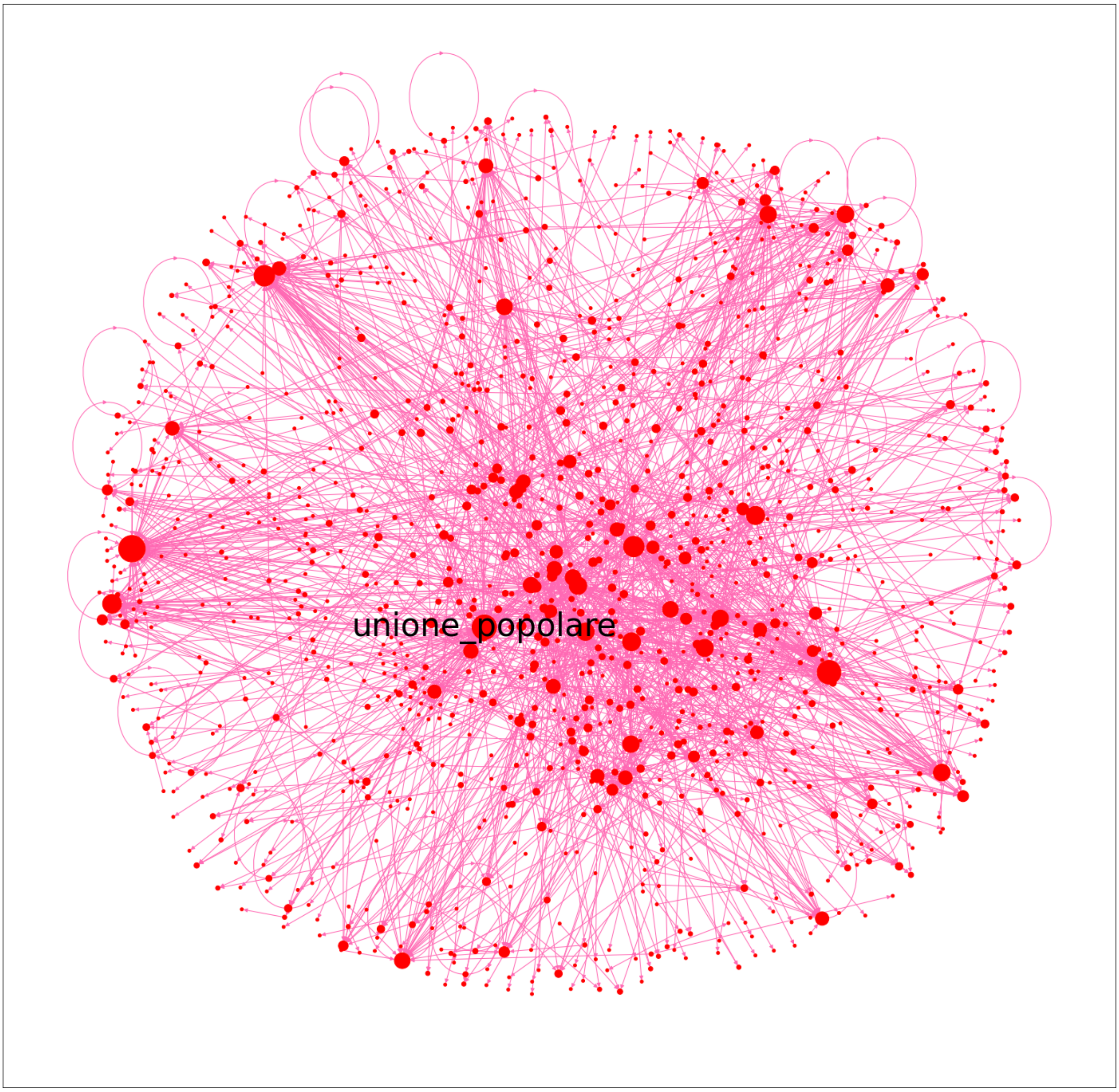

Figure 5: Plot of the Unione Popolare community. Vertex size is proportional to its in-degree, named accounts are those among the 10 with highest in-degree which are verified and belong to official party organs or affiliated politicians.

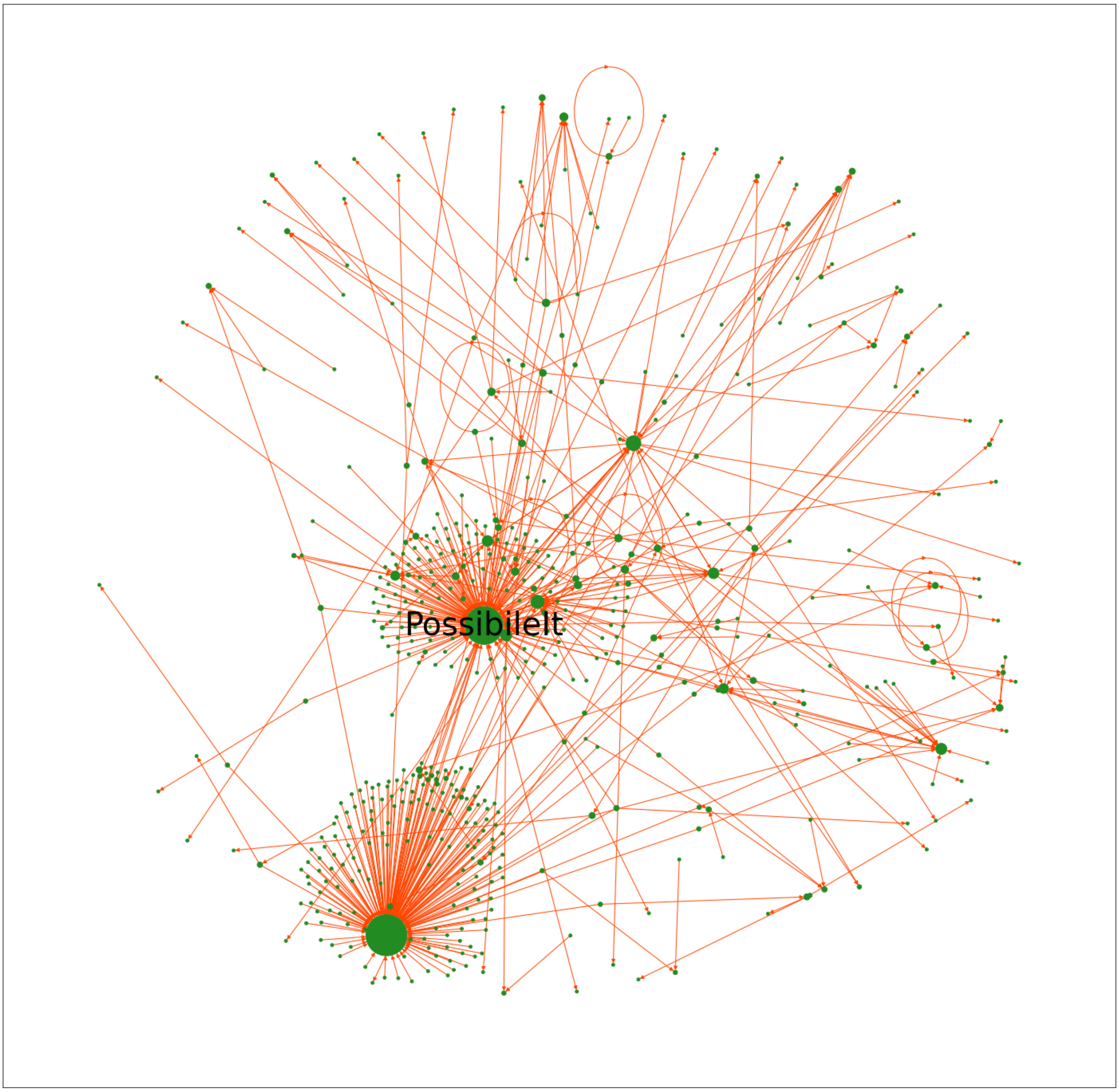

Figure 6: Plot of the Sinistra Italiana & Verdi community. Vertex size is proportional to its in-degree, named accounts are those among the 10 with highest in-degree which are verified and belong to official party organs or affiliated politicians.

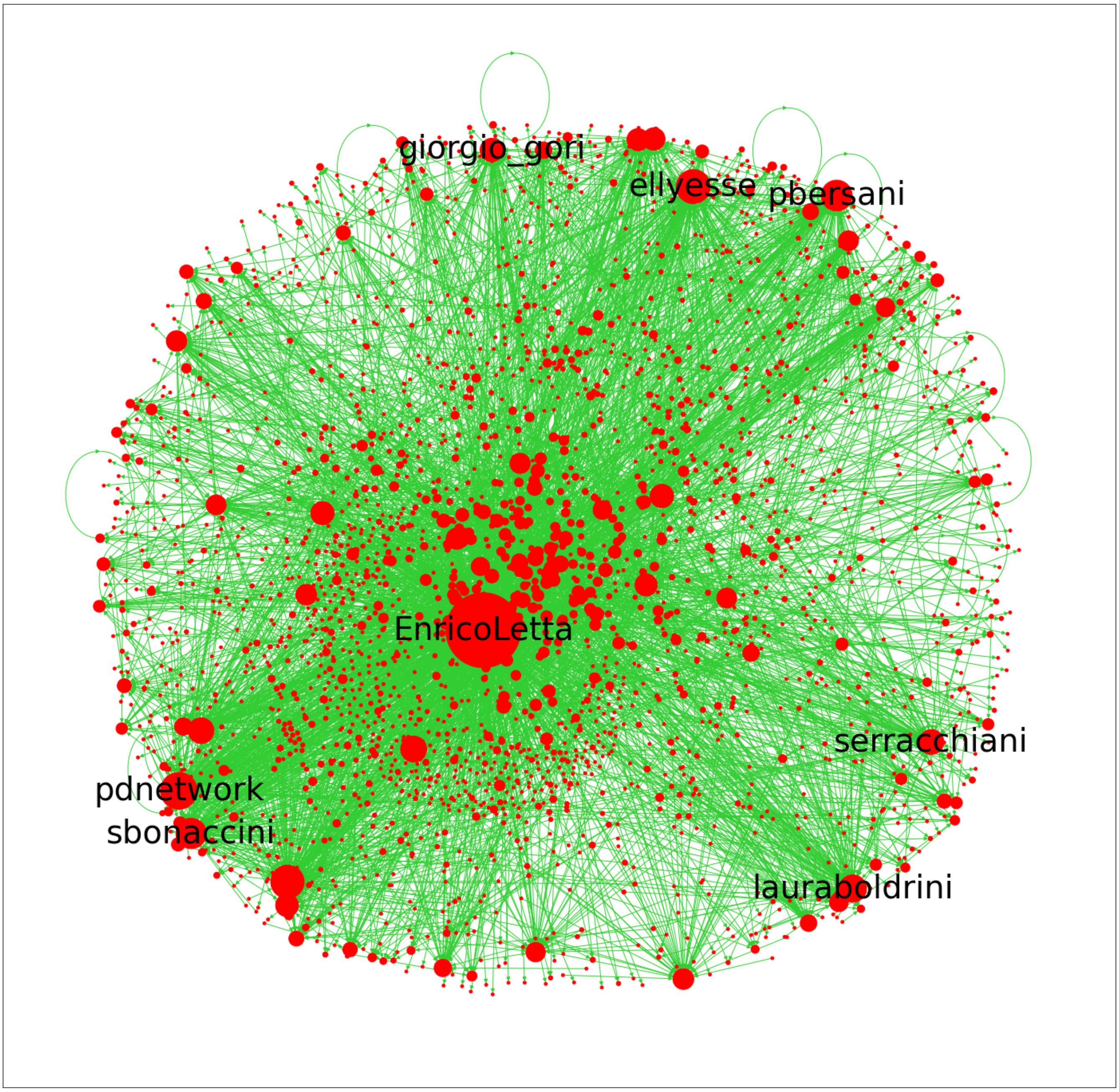

Figure 7: Plot of the Partito Democratico community. Vertex size is proportional to its in-degree, named accounts are those among the 10 with highest in-degree which are verified and belong to official party organs or affiliated politicians.

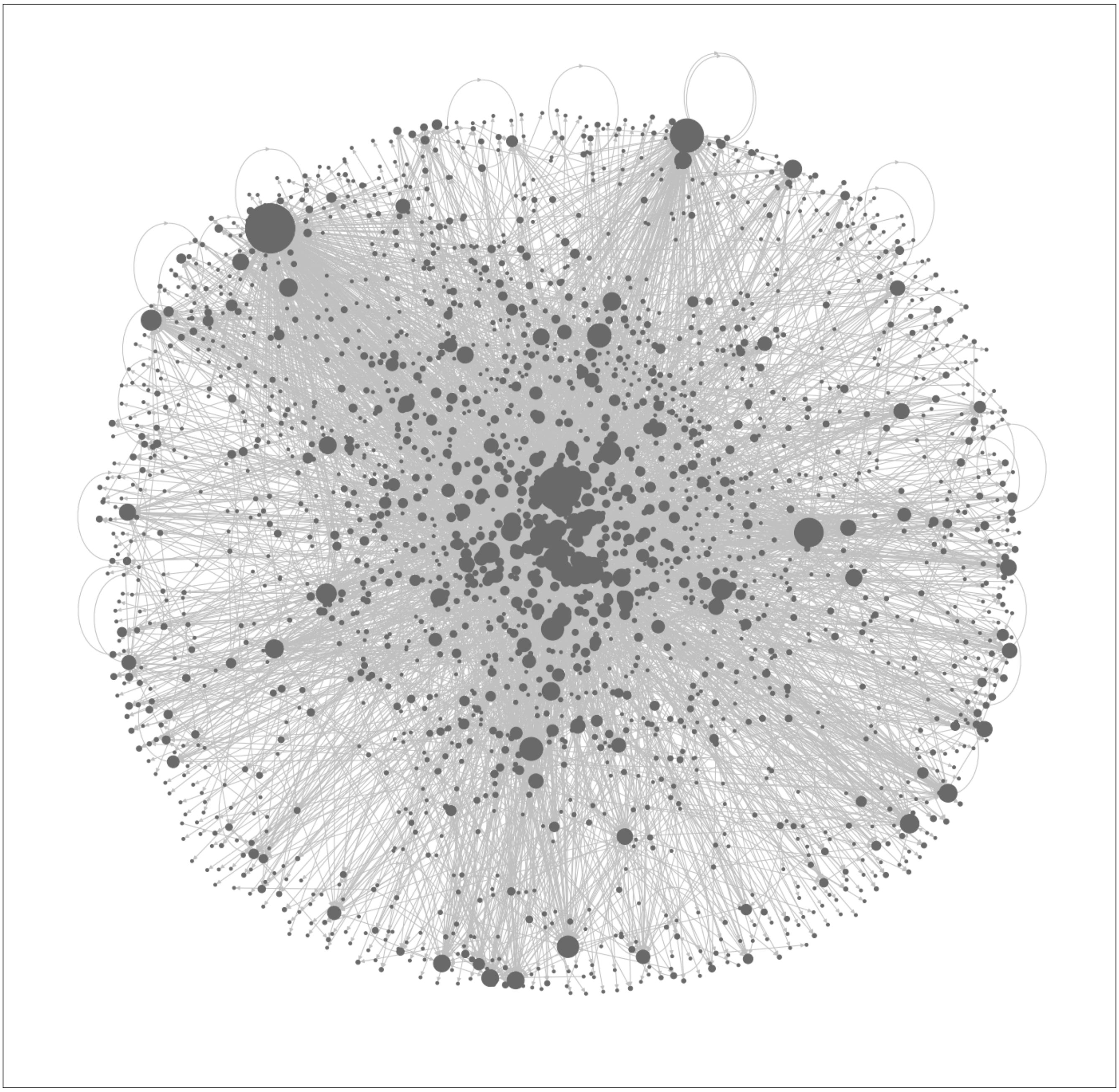

Figure 8: Plot of the Far Right community. Vertex size is proportional to its in-degree, named accounts are those among the 10 with highest in-degree which are verified and belong to official party organs or affiliated politicians.
